# A model-based optimization framework for the inference of regulatory interactions using time-course DNA microarray expression data

**DOI:** 10.1186/1471-2105-8-228

**Published:** 2007-06-29

**Authors:** Reuben Thomas, Carlos J Paredes, Sanjay Mehrotra, Vassily Hatzimanikatis, Eleftherios T Papoutsakis

**Affiliations:** 1 Laboratory of Molecular Toxicology, National Institute of Environmental Health Sciences, National Institutes of Health, Research Triangle Park, North Carolina, USA; 2 Department of Industrial Engineering and Management Science, Northwestern University, Evanston, Illinois 60208-3120, USA; 3 Laboratory of Computational Systems Biotechnology, EPFL, CH-1015 Lausanne, Switzerland; 4 Department of Chemical and Biological Engineering, Northwestern University, Evanston, Illinois 60208-3120, USA; 5 Gevo, Inc., 133 N. Altadena Dr. Suite 310, Pasadena, CA 91107, USA; 6 Dept. of Chemical Engineering and the Delaware Biotechnology Institute, University of Delaware, Newark, DE 19711, USA

## Abstract

**Background:**

Proteins are the primary regulatory agents of transcription even though mRNA expression data alone, from systems like DNA microarrays, are widely used. In addition, the regulation process in genetic systems is inherently non-linear in nature, and most studies employ a time-course analysis of mRNA expression. These considerations should be taken into account in the development of methods for the inference of regulatory interactions in genetic networks.

**Results:**

We use an S-system based model for the transcription and translation process. We propose an optimization-based regulatory network inference approach that uses time-varying data from DNA microarray analysis. Currently, this seems to be the only model-based method that can be used for the analysis of time-course "relative" expressions (expression ratios). We perform an analysis of the dynamic behavior of the system when the number of experimental samples available is varied, when there are different levels of noise in the data and when there are genes that are not considered by the experimenter. Our studies show that the principal factor affecting the ability of a method to infer interactions correctly is the similarity in the time profiles of some or all the genes. The less similar the profiles are to each other the easier it is to infer the interactions. We propose a heuristic method for resolving networks and show that it displays reasonable performance on a synthetic network. Finally, we validate our approach using real experimental data for a chosen subset of genes involved in the sporulation cascade of *Bacillus anthracis*. We show that the method captures most of the important known interactions between the chosen genes.

**Conclusion:**

The performance of any inference method for regulatory interactions between genes depends on the noise in the data, the existence of unknown genes affecting the network genes, and the similarity in the time profiles of some or all genes. Though subject to these issues, the inference method proposed in this paper would be useful because of its ability to infer important interactions, the fact that it can be used with time-course DNA microarray data and because it is based on a non-linear model of the process that explicitly accounts for the regulatory role of proteins.

## 1. Background

Inference of regulatory interactions in a genetic system provides fundamental biological knowledge and significant efforts have been invested for the solution of this problem, [1-23]. The method we propose in this paper improves upon the previous contributions to the solution of this problem: it employs a more realistic model, it reduces the effect of noise on the solution obtained, it avoids the costly step involving numerical integration and, significantly, it explicitly utilizes gene expression ratios, which are typically the primary data of microarray-based gene expression analysis. Here, we use an S-system based [24-29] model that explicitly accounts for proteins serving as regulatory agents. It also accounts for the non-linear dependency of transcription rates in the protein concentrations. We are solely dealing with gene expression data in view of the fact that reasonably-complete proteomic data are not readily available. We used the same model as in our previous work, [12] for the development of a method for gene regulatory network inference based on steady state gene expression ratio data. In this paper, a heuristic solution for the problem is given, as dictated by the S-system based model and time-varying gene expression ratio data. The computational complexity of the method is exponential in the number of genes in the system. However if a subset of the interactions were already known to exist, then the method could be used on networks with a larger number of genes. The impact of noise in the data is reduced by using smoothing splines as approximations to the time profiles of gene expression.

The model used in this paper shares similarity with inference methods based on S-system models [11-15]. However, these earlier methods do not consider the effect of proteins (whose concentrations are not measured) in regulating gene expression. Also, every evaluation of the objective function set up in [11] and [13] for optimization required the integration of a set of differential equations. This integration can be costly in terms of computational resources, as was pointed out in [28] and [29].

Related to the methods based on the S-system models are methods based on linear differential equations [16-19]. The methods of Refs. [17] and [19] involve a least square fitting approach, but their models do not involve protein concentrations. Dasika *et al*. [2] used a linear regulatory model but allowed the current gene expressions to depend on the levels of gene expression of the previous time points. This time delay of the action of an mRNA on the transcription rates may capture the delay due to the protein-translation process and possible protein modification events like glycosylation, phosphorylation, methylation etc. However, the value of the time-delay parameter cannot be mapped easily to the biophysical and biochemical process it represents. The model presented here directly accounts for the protein translation process and thus there is an implicit time-delay in the regulation of gene expression. The model used in Ref. [18] involves both mRNA and protein concentrations. However, the authors assume that all protein concentrations can be measured. The work in Refs. [20-23] are representative of methods which analyze the time course gene expression data using a Bayesian network framework. This framework assumes a linear model between gene-expression levels at multiple time points and hence is similar, conceptually, to the one used in Ref. [16].

Most of the previous model-based methods (Eg. [11,13,16,19]) assume that the gene-expression data are available as absolute concentrations and they also assume linear, additive action of the regulatory mRNAs on the transcription rates. The method presented here is tailored for the analysis of *relative *gene expression data, and it can be regarded as a non-lineargeneralization of the previous models. Apart from these models, there are model-based identification methods that include even broader description of cellular processes by including models for metabolic processes [14]. However, the applicability of such models is restricted to smaller systems because of the complexity involved due to experimental measurements and computational requirements.

Here we describe a model-based inference approach of the regulatory network of a genetic system using time-varying mRNA-expression ratios obtained from experiments involving DNA microarrays. We employ an S-system approach to model the transcription and translation processes and, propose an optimization-based regulatory network inference method. The method is tested using synthetic data from a model genetic network of genes, and is applied on expression data of a core subset of genes involved in the sporulation cascade of the prokaryote *Bacillus anthracis*.

## 2. Results

### 2.1 Dynamic regulation model and its characteristics

According to the S-system based model of gene expression and protein synthesis [12] the mass balances (rates of change of concentrations) for each mRNA *i*, *m*_*i*_(*t*), and protein *i*, *p*_*i*_(*t*), in a system of *n *genes are described by the following equations,



where *V*_*sm*,*i *_and *V*_*dm*,*i *_denote the rates of synthesis and degradation rates of the *i*^*th *^mRNA, *V*_*sp*,*i *_and *V*_*dp*,*i *_denote the rates of synthesis and degradation rates of the *i*^*th *^protein, *α*_*i *_and *γ*_*i *_denote the transcription and translation rate constants, and *β*_*i *_and *δ*_*i *_are the first-order decay constants of the mRNA and protein, respectively. The real parameters ***ε***_ig _quantify the strength of regulatory control exerted by the activity of protein *g *on the synthesis rate of mRNA *i*. If ***ε***_ig _is equal to zero, protein *g *does not affect the expression of gene *i*, and if ***ε***_ig _is positive (negative), then protein *g *induces (represses) the expression of gene *i*. A discussion on the ranges of these parameters can be found Appendix 1 of [12] and [29].

### 2.2 Derivation of the optimization method

The basic goal is to quantify the strengths of regulatory interactions and rate constants that best fit the dynamic model described by Equation (1) to a given set of time-course, gene-expression data. We consider a network of *n *genes, which are perturbed at some time before *t *= 0, from *t *= 0 onwards there are no external perturbations, and the mRNA and protein concentrations change continuously over time.

Experimental methods like the DNA microarrays typically measure the absolute value or the logarithm of gene (mRNA) expression ratios at discrete points in time. Thus, the log-expression ratio for gene *i *at time *t*_*j *_is given by,



where  is a reference state for gene *i*.

Protein concentrations are not directly observable, unless an accurate proteomics technology is used [31,32], and therefore we employ the following novel methodology that utilizes smoothing cubic splines, [33]. We fit smoothing splines through the gene expression ratios at different time points and use them to predict the protein concentrations. It is analytically possible to do this because of the polynomial forms of the splines. As a result, we can avoid the expensive steps of numerical integration during the parameter estimation stage. The concentration of protein *i *at time *t *can be written in the following form:

*p*_*i*_(*t*) = *p*_*i*_(0)*f*_*i*_(*δ*_*i*_, *t*) + *γ*_*i*_*h*_*i*_(*δ*_*i*_, *t*)

where *p*_*i*_(0) is the initial concentration of protein *i*, *f*_*i*_, and *h*_*i *_are non-linear functions of *δ*_*i *_and time, *t*, derived using the splines fitted to the gene-expression data in the mass balance equations for the proteins. The initial protein concentrations *p*_*i*_(0) and the reference states  are also unknown parameters. Estimates of the decay constants *β*_*i *_and *δ*_*i *_can be obtained from the available half-life of mRNAs and proteins [34,35] and also see Section A6.2, Additional file. In the following analysis, we will assume that these decay constants are known. The derivation of Equation (2) is given in the Section 4.1.

We can now estimate the unknown parameters of the network by solving the following nonlinear mixed-integer mathematical programming problem:



where,



subject to

-*DY*_*ij *_≤ *ε*_*ij *_≤ *DY*_*ij*_, *i*, *j *= 1,2,...,*n*

*Y*_*ij *_∈ {0,1}, *i*, *j *= 1,2,...,*n*

*α*_*i*_, *γ*_*i*_, , *P*_*i*_(0) ≥ 0, *i *= 1,2,...,*n*

where,  is the error term which is an approximate restatement of the mass balance equations of all the *n *genes at *N*_*t *_discrete points in time (see Section 4.2 for the derivation of this term).  represents the vector of regulatory interactions affecting gene *i *and |||| represents its Euclidean norm. *τ*_*i *_is a regularization parameter for each gene *i*. Regularization [36] of the formulation can be used when the quality of the time-series data leads to ill-conditioned systems. Regularization has been also used in the network inference method proposed by Kikuchi *et al*. [13], and by Gardner *et al*. [3] in the form of ridge regression. However, if the data do not lead to an ill conditioned system, such regularization is not necessary and the regularization parameters *τ*_*i *_are set equal to zero. Therefore, the objective in Equation (3) minimizes the sum of the error in fitting the model to the experimental data, and the weighted norm of the strength of the interactions.

*Y*_*ij *_is a binary variable which is equal to 1 when gene *j *interacts with gene *i *or zero otherwise. *D *in Equation (5) is some positive number that limits the strength of an interaction. This constant can either be assigned a number based on prior biological knowledge (for typical kinetics, it can be set equal to one for Michaelis-Menten kinetics, or up to 4 (for the usual tetramer-dependent cooperative kinetics) or set equal to an arbitrarily large number. Constraints (5), (6) and (7) enforce the assumption that each gene is regulated by not more than *k *other genes as has been explained in [16] and [12]. Constraint (8) guarantees the non-negativity requirements of the other unknowns.

### 2.3 Coordinate descent based heuristic method

There are two main issues associated with computing the solution of the optimization problem described by Equations (3)-(8). First, the objective function in (3) is convex in the terms of strength of interactions, *ε*_*ij *_and in the logarithm of the transcription rates, log(*α*_*i*_), and it is non-convex in the translation rate constants, *γ*_*i*_, the initial protein concentrations, *p*_*i*_(0) and the reference mRNA expression states, . In general globally optimal solutions can be found only for convex optimization problems [37,38]. A second issue arises from the large number of continuous and discrete variables (or unknown parameters) involved in each optimization since the time for solving such problems increases exponentially with the number of variables. In order to address these two issues, we introduce a coordinate-descent based heuristic method to solve the inference problem. The method is based on the observation that the three sets of parameters, *γ*_*i*_, , and *p*_*i*_(0), link all the genes together through the objective function, in the sense that if these three sets of parameters were known, then the resulting optimization problem would be convex in its unknowns and the problem could be equivalently split into *n *sub-problems, one problem for each gene. Thus, instead of dealing with one mixed-integer optimization problem of O(*n*^2^) variables, we would have *n *mixed-integer problems with O(*n*) variables. The method then essentially repeats the two steps below for a given number of times (say *N*_*l*_),.

1. Fix the values of *γ*_*i*_, , and *p*_*i*_(0), as determined either by an initial guess or from Step 2 below. Solve *n *(mixed-integer quadratic) optimization problems, one for each gene *i*, in the parameters  and log(*α*_*i*_). Each problem is mathematically stated as:



subject to

-*DY*_*ij *_≤ *ε*_*ij *_≤ *DY*_*ij*_, *j *= 1,2,...,*n*

*Y*_*ij *_∈ {0,1},i, *j *= 1,2,...,*n*

log(*α*_*i*_) ≥ -*A*

where *A *is some large positive number.

2. Fix the values of  and log(*α*_*i*_) determined from Step 1 and solve the following optimization problem in the three sets of parameters, *γ*_*i*_, , and *p*_*i*_(0).

min 

subject to,

*γ*_*i*_, , *p*_*i*_(0) ≥ 0, *i *= 1,2,...,*n*

Our numerical studies suggest that the improvements attained by increasing the number of repetitions of the above two steps are marginal (Figure A.1, Additional file), i.e., a relatively small value for *N*_*l *_may be good enough.

Different initial guesses would potentially lead to different solutions, and the proposed method does not guarantee finding the globally optimal solutions. A procedure of reporting the *best solution *considers the network of interactions derived from all the collected solutions, i.e., of similar optimum objective function values, by accepting an interaction to be present if it is inferred to be present in the majority of the solutions. Since the set of optimal solutions can be considered as alternative networks that are consistent with the experimental data, an interaction can be considered physiologically significant if it occurred in the majority of the solutions.

### 2.4 Parameters and issues affecting the performance of the algorithm

The applicability of any genetic network inference method is affected by a number of factors (see Ref. [39] for a mathematical description of such factors). We used 10-gene synthetic networks (see Section A3, Additional file) to generate data that are used to study the performance of the algorithm. The following six factors which are known to have an effect on the performance of inference algorithms were studied: (i) the degree of similarity between the time profiles of the expression of different genes; (ii) the number of experimental samples available; (iii) noise in the data; (iv) interactions involving genes that do not show significant variation; (v) the parameters *N*_*l *_(the number of iterations), and *N*_*t *_(the number of discretization time points in the objective function) of the heuristic method; and (vi) missing genes from the analysis.

The time-series data were obtained by the integration of the S-system of differential equations (1) in MATLAB [40], for different values of the parameters. The mixed-integer non-linear solver of LINDO [41] was used in all the optimization problems. In all the numerical studies in this section only, we assume that the parameters *γ*_*i*_, , *p*_*i*_(0) are known. This will allow us to correctly base our conclusions using globally optimal solutions.

The degree of similarity between the different time profiles of mRNA expression is an important determinant of the amount of information present in the data. We studied three different types of networks that we labeled "Low", "Medium" and "High" according to the degree of similarity between the different profiles which we quantified using the condition number of the matrix Φ formed by the logarithm of protein concentrations at each time point:

Φ = [log(*p*_*gj*_)], *g *= 1,...,*n*, *j *= 1,2,...,*N*_*t*_

**Figure 1:**
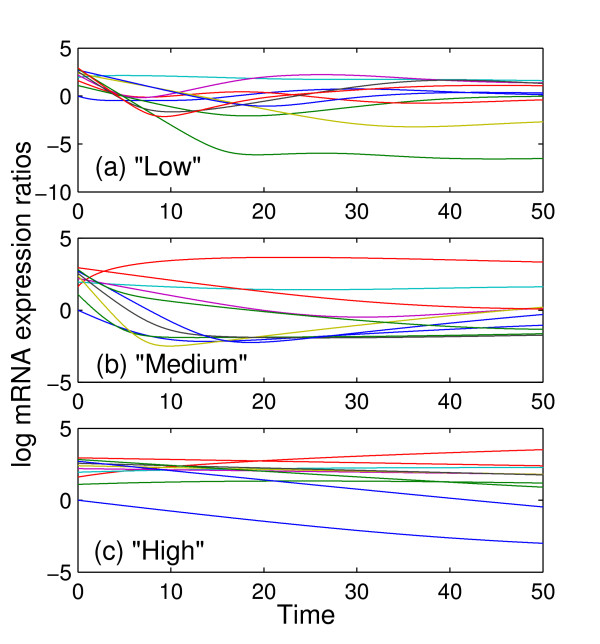
**Time profiles of log mRNA expression ratios for three representative synthetic networks**. Logarithm of mRNA expression ratios as a function of time for three networks. The network in (a), "Low" results in a relatively lower degree of similarity between the different gene-expression patterns in the system, the network in (b), "Medium" in a medium degree of similarity, while the network in (c), "High" results in a relatively high degree of similarity. The units of time are arbitrary but are consistent with the units of the parameters of the system.

The time series of the logarithm of expression ratios are shown in Figure 1, for the three types of networks: a network with condition number of ~10^3 ^("Low"; Figure 1(a)), a network with condition number ~10^6 ^("Medium"; Figure 1(b)) and that in Figure 1(c) in a condition number ~10^9 ^("High").

**Figure 2:**
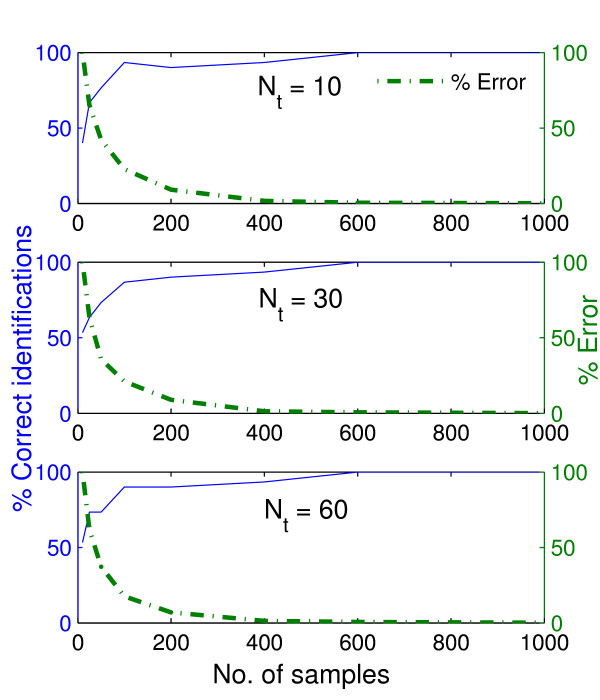
**Variation of correct identifications and identification errors with experimental samples and discretizations for "Low" network**. Variation of the percentage of correctly identified interactions among 30 known interactions and the error as a percentage of the error obtained with the smallest number of samples. The variations are with respect to the number of experimental samples chosen and the number of discretizations, ***N***_*t*_. The "experimental" data are obtained by simulation using the "Low" synthetic network (see Figure 1).

**Figure 3:**
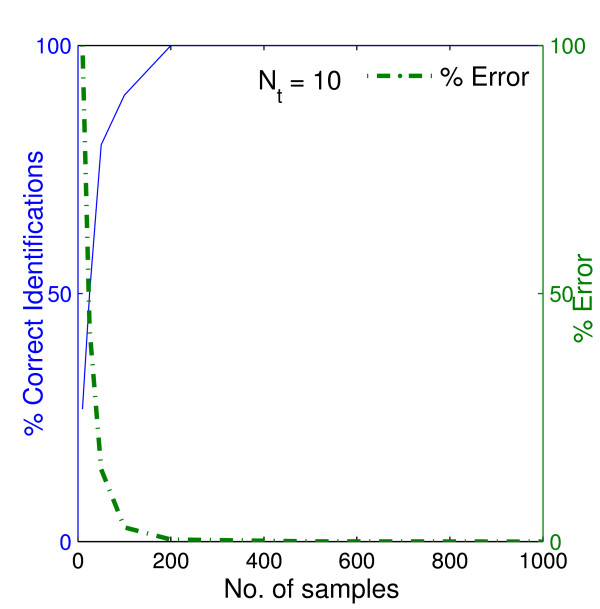
**Variation of correct identifications and identification errors with experimental samples for "Medium" network**. Variation of the percentage of correctly identified interactions among 30 known interactionsand the error as a percentage of the error obtained with the smallest number of samples. The variations are with respect to the number of experimental samples chosen. The "experimental" data are obtained from simulations using the "Medium" synthetic network of Figure 1. 10 time points were used in the optimization method.

As expected, the performance of the method, with respect to percentage of the correct identifications, improved as the number of uniformly spaced experimental samples, *N*_*s*_, used for the protein estimation increased (Figure 2) since their size affects how much of the true variation of the time profiles are captured in the data. Note that the percentage of correct identifications refers to the percentage of true positives (interactions) among all the positives (true and false) identified by the method. The parameter *k *was set to 3 and hence the total (true and false) positives equal to 30. The performance of the method is not very sensitive to *N*_*t*_. Moreover, most of the interactions in the network appear to be quite robust to sampling frequency. For example at least 50% of the interactions were correctly identified for all sample sizes (Figure 2). As the condition number of the data increases, i.e., the profiles exhibit lesser variation, the number of samples required for correct identification is reduced (Figure 3).

We also studied the effect of noise in the data of expression ratios using the "Low" network with 1000 "experimental" samples. We chose a large number of samples in order to avoid bias in the results due to a sampling error. We used smoothing splines that are known to provide a good compromise between the approximation error and the smoothness of the resulting curve [33]. A parameter called the smoothing parameter controls the degree of smoothness of the spline, and as expected the choice of the value of this parameter will have an impact on the performance of the method. We used the technique of Generalized Cross Validation [42], which has been shown to provide good estimates for the smoothing parameter, as implemented in the R statistical package [43]. We found that about 50% of the interactions are very sensitive to noise, while even with a 50% error in the data, we are able to infer about 30% of the interactions (Table 1).

**Table 1:** Variation of correct identifications with increasing levels of noise in data

% Error	% Correct
0.01	70.8 ± 0.2
s1	44.1 ± 0.2
5	41.4 ± 0.2
10	38.2 ± 0.2
20	34.8 ± 0.2
50	31.9 ± 0.2

Variation of the percentage of correctly identified interactions (given at a 95% confidence interval) with the percentage error in the data. E.g., a 5% error implies that all the experimental expression ratio data are known only with an error of ±5%. The "experimental" data are obtained from simulations using the "Low" synthetic network (Figure 1).

The DNA microarray technology tends to suppress the measured expression ratios [44], and some of the gene expression profiles do not show much variation, i.e., they are more or less constant over the period of observation. Therefore, it is unlikely that the algorithm, due to numerical constraints, will infer interactions involving these genes, since these interactions will be absorbed in the parameter *α *that quantifies the transcription rate constant. Therefore, in order to make the experimental gene-expression profiles more suitable for the genetic-network inference method, we examined the possibility of rescaling all logarithmic expression ratios by a constant factor or raise all expression ratios to a certain power. This way the larger expression ratios (>1) become larger while the smaller expression ratios (<1) become smaller.

If all the interacting genes in a network are not considered for analysis by an inference method, then incorrect interactions are likely to be identified. If we remove genes that contribute to significant regulatory interactions, the number of incorrect identifications would increase (see Table A.7, Additional file). Finally, relatively small errors in the estimates of the half-lives of the mRNAs and proteins cause only a modest deterioration in the performance of the method (see Table A.8, Additional file).

### 2.5 Algorithm testing

#### 2.5.1 Synthetic networks

The algorithm was tested using data from the "Low" network with 1000 "experimental" samples. There was no noise in the data but the parameters *γ*_*i*_, , *p*_*i*_(0) were now assumed to be unknown, i.e., the problem was *non-convex*. We found 7 solutions using the Coordinate descent heuristic method starting from 7 random initial guesses and all solutions converged to similar (O(1)) objective function values. The best solution was identified as the one whose interactions occurred in the majority of the solutions. The '4 out of 7' solution identified 11/30 interactions correctly while the '5 out of 7' solution identified 10/30 interactions correctly. If the method identifying the interactions were random, and since we have assumed a 10-gene network with 3 regulatory inputs for each gene, an interaction will be identified as inducing with probability 15%, repressing with probability 15% and absent with probability 70%. Therefore the average number of correct interactions identified will be 15% (4.5/30), suggesting that the heuristic method we are using is doing better than a random method.

Note that we found that about 30% of the interactions could be identified with a large amount of noise, or when the parameters *γ*_*i*_, , *p*_*i*_(0) are unknown. The results here give a similar coverage indicating that these 10/30 interactions are not just robust to noise but are also important in the sense that they are captured in all the solutions found.

To give an estimate of the time required to obtain a solution, it took about 15 hours to obtain 5 solutions, each running in parallel on a P4, 2 GHz, and 1 Gig RAM PC. Since the main emphasis of this study was not to obtain a computationally efficient method, only this estimate of the time taken for a solution is provided. Note that the computational complexity of the heuristic method is of O(*n*e^*n*^).

#### 2.5.2 A network from the sporulation cascade of *Bacillus anthracis*

##### 2.5.2.1 Background

*Bacillus anthracis *is an endospore-forming bacterium (a prokaryote) that is responsible for the anthrax disease. Under environmental-stress conditions, like most bacilli, it commits to sporulation via the bacillus endospore program. Mature spores can survive many extreme conditions, thus assuring species survival. When conditions are suitable, the endospore germinates and the organism then can begin to grow again.

##### 2.5.2.2 Data and choice of genes

**Figure 4:**
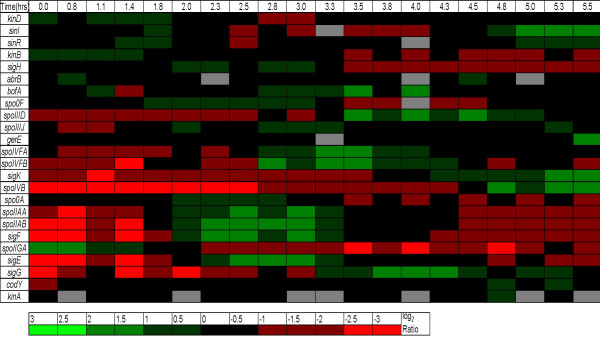
**Time-course variation of subset of important genes in the sporulation cascade of *B. Anthracis***. The time-course (in hours) variation of the logarithm of expression ratios in color-coded format (green indicates up-regulation, red indicates down-regulation, grey indicates missing data and the intensity of the color indicates the level of regulation) of 24 important genes in the sporulation cascade of *B. anthracis *([29]). An approximate measure of the intensity of the color to the magnitude of the log_2 _ratio (fold change) is also given. Note that the ratio refers to that of the actual expression value to the expression value at the reference state.

Liu *et al*. [45] performed genome-scale DNA microarray analysis of a sporulating batch culture of *B. anthracis*, and they monitored the expression ratios of over 2000 genes at 20 points over a time course of about 6 hours. Figure 4 shows the logarithm values of expression ratios of 24 of the important players in the sporulation cascade [45]. The procedure of expression data retrieval and the choice of the 24 players are further elaborated in Section A5.1, Additional file. From these, we excluded a subset of genes based on the following considerations:

• *kinA *had an insufficient number of usable data points.

• Genes like *kinD*, *abrB*, codY exhibited insufficient variation.

• The transcriptional and total protein levels of Spo0A are not relevant; rather, it is its activation (phosphorylated Spo0A, Spo0A~P) that matters, and, in the absence of reliable *kinA *data, this is better captured by the expression of *spo0F*. Thus, we will use *spo0F *expression to represent Spo0A~P and is shown below as *spo0F*/Spo0A~P.

• Genes with similar profiles were not considered. E.g. *spoIIAA *and *spoIIAB *had similar profiles to *sigF *and *sigE*. *spoIVB *had a similar profile to *sigK*.

• The variation of *spoIIGA *did not correspond to what is known about its role in the cascade. From prior biological knowledge [46], one would have expected *spoIIGA *to have a profile similar to those of *sigF *and *sigE*.

The chosen subset of 9 genes consists of *spo0F/*Spo0A~P, *sigF*, *sigE*, *spoIIIJ*, *sigG*, *spoIVFB*, *spoIIID*, *sigK *and *gerE*. Refer to Section A5.2, Additional file for a discussion of the biological basis of this choice.

##### 2.5.2.3 Inferred interactions

In the experiments [45] to generate the *B. anthracis *microarray data, the reference state (parameter , in terms of copies of mRNA per cell) for the expression of gene *i *was taken to be the average of equal amounts of samples drawn at each of the time points over the course of the experiment. If a gene was expressed only for a short period of time, then the expression ratios during this period would be relatively high. But if the expression of a gene changed slightly over the entire course of the experiment, then the expression ratios would show only very small variations around the value of 1. Because of this and because, as stated, DNA-microarray analysis underestimates the true expression ratios, the log-expression ratio data were scaled by a factor of 2 (see Section 2.4) in order to accentuate the variations within each profile. Smoothing splines were fit to the expression ratio data derived from these scaled log-expression ratio ones. The graphs of these smoothing splines along with the units and the bounds on the different unknown parameters involved in the optimization problem are given in Section A6, Additional file.

The interactions that were identified in at least 5 out of the 7 solutions are given in Table 2. We observe that many important known interactions are captured. These include the effect of: *spo0F*/Spo0A~P on *sigF*, *sigF *on *sigE*, *sigE *on *sigG*, *spoIVFB *on *sigK*, and *sigK *on *gerE*. Also note that several inhibiting interactions that were identified are not known to exist. This could be because the set of genes that were considered did not include genes or conditions necessary to shut down the genes under consideration. Hence the algorithm picked genes whose profiles were probably closest to the ones that the true inhibiting genes/conditions would possess. Also note that the set of genes did not involve a gene (like *kinA*) or condition (e.g., starvation) that would initiate the activation of Spo0F and eventually that of Spo0A. So the fact that *gerE *was identified as being responsible for the activation of *Spo0A *can be viewed as a numerical artifact that reduces the objective function value the most when compared with the reduction obtained when other genes serving as activators of *Spo0A*. A more detailed discussion of the results is presented in Section A7, Additional file.

**Table 2:** Identified interactions among subset of genes in *B. anthracis*

	*spo0F/*Spo0A~P	*sigF*	*sigE*	*spoIIIJ*	*sigG*	*spoIVFB*	*spoIIID*	*sigK*	*gerE*
spo0F/Spo0A~P	0	0	0	*	0	0	-1	0	1
*SigF*	1	0	0	0	0	*	-1	0	0
*SigE*	0	1	0	*	*	0	*	0	0
*SpoIIIJ*	1	0	0	0	*	0	*	*	0
*SigG*	-1	0	1	0	0	0	0	0	*
*spoIVFB*	*	0	1	0	0	0	*	-1	0
*SpoIIID*	-1	1	1	0	0	0	0	0	0
*SigK*	*	0	0	*	0	1	0	1	0
*GerE*	0	0	0	0	-1	-1	0	1	0

Identified interactions obtained from the inference method for the set of 9 genes involved in the sporulation cascade on *B. anthracis*. '1' indicates an activating interaction, '-1' an inhibiting interaction, '0' an absent interaction and '*' an interaction for which a conclusion could not be drawn. A row corresponds to a regulated gene and a column corresponds to a regulator gene. For example, *sigE *positively regulates *sigG*, *spoIVFB *and *spoIIID*.

Overall, the algorithm was able to identify many important interactions based on this set of experimental data. While we can assess the effect of all the factors discussed in Section 2.4 on the specific set of experimental data, we propose that the missing genes/signals are probably mainly responsible for the incorrect interaction identifications for the 'start' gene (*Spo0A*) and for those responsible for shutting down the expression of various genes.

## 3. Conclusion

We have developed a regulatory inference method that can be used on dynamic, time-course expression data such as those obtained from DNA microarray analysis. The method takes into account the non-linear regulatory roles of the corresponding proteins in the system. We validated our approach on a synthetic network and on a set of genes that are involved in the sporulation cascade of *B. anthracis*. We did not consider the impact of external perturbations during the course of the experiment. However, the extension of our approach to include this case would be straightforward if we assume that the external perturbations can be modeled as artificial genes that are not influenced by any of the genes involved in the study.

The ability of the method to generate a set of alternative regulatory networks that are consistent with the experimental data allows a broader analysis of a system when the number of experimental samples is low and the degree of similarity between the time profiles of different genes is high.

## 4. Methods

### 4.1 Prediction of protein concentrations

Let *N*_*s *_denote the number of time points at which log-expression ratios are measured, and these points are denoted as {*t*_1_, *t*_2_,…} and *t*_1 _= 0 and  = *T*. For each gene *i*, we perform a cubic spline interpolation [33] through the points (*t*_*j*_, ) for *j *in {1..***N***_*s*_}. This results in the following *n*(*N*_*s*_-1) cubic polynomials,



So  represents a polynomial approximation to *m*_*i*_(*t*) in the interval [*t*_*j*_, *t*_*j*+1_]. Using this approximation, the general solution to the protein mass-balance equation can be approximated by,



where the first term represents the homogeneous solution to the protein mass-balance differential equation and the second term the particular solution. The values for *b*_*ij *_are given by,



This can be verified by checking that the particular solution indeed satisfies the protein mass-balance equation. *c*_*ij *_can be obtained in terms of the initial protein concentration, *p*_*i*_(*t*_1_) (*t*_1 _= 0),  and *γ*_*i *_by enforcing the continuity of the protein function across the break points, i.e.,



We can then show that



where *Q*_*i*_(*t*) and *R*_*ij*_(*t*) are defined in terms of , {*t*_*q*_}, for *q *in {1..*j*}, *d *in {1..4} and *δ*_*i*_. Let,



Then,



So now we have approximations to *M*_*i*_(*t*) and *p*_i_(*t*) for any time *t *in the interval [0, *T*]. The error in the approximation of *M*_*i*_(*t*) is O(*T *× *N*_s_^-4^) [33] while the error in approximation of *p*_i_(*t*) is the sum O(*γ *× *T *× *N*_s_^-4^) and the error in the estimation of the initial protein concentration, see Section A2, Additional file.

### 4.2 Derivation of objective function for optimization problem

This section describes the derivation of the error term in the objective function (Equation (3)) for the optimization problem of the inference method. The mass balance equation for any gene *i*, at any time *t*, is given by (from Equation (1)),



If both the left-hand side and right-hand side of the above equation are non-zero then,



The last equation is a function of time *t*, that is exactly equal to zero over the entire time per period of observation, [0, *T*]. Therefore the integral of this function with respect to time *t*, over this time period should also be zero.



The above equation should hold for all the *n *genes in the system. Hence,



The objective function can further be simplified by approximating the integral by a discrete summation, say at *N*_*t *_points. In other words, we require that the mass balance equations are satisfied only at a finite number of points as opposed to every time point in the period of observation. Note that this discrete summation can also be viewed as a trapezoidal rule-based approximation of the integral:



## Authors' contributions

RT proposed the identification method, did the analysis and prepared the manuscript. CJP assisted in the identification of the *B. anthracis *network, curated the experimental data for use by the identification algorithm and provided invaluable biological insights and literature information. ETP and VH identified the general problem and provided the overall project direction. VH suggested and developed the modeling framework for the analysis and oversaw the detailed model development effort. ETP oversaw the manuscript preparation and editing, and provided guidance on biological issues and their interplay with computational issues. SM advised on and checked the model development and optimization formulations. All authors have read and approved the final manuscript.

## Supplementary Material

Additional file 1 Additional derivations, data and results. This document has information on derivations, explanations and data that is related to the work in this paper. However knowledge of this information is not crucial to understanding what is stated in the paper. For the interested reader, the paper does refer to this material at appropriate places in the paper. Click here for file
