# Supplementary material for: A model-based optimization framework for the inference of regulatory interactions using time-course DNA microarray expression data
Source: BMC Bioinformatics. 2007 Jun 29;8:228. doi: 10.1186/1471-2105-8-228 (PMC1940027; doi:10.1186/1471-2105-8-228)
Supplement: Additional file 1 — Additional derivations, data and results. This document has information on derivations, explanations and data that is related to the work in this paper. However knowledge of this information is not crucial to understanding what is stated in the paper. For the interested reader, the paper does refer to this material at appropriate places in the paper. [file 1471-2105-8-228-S1.doc]

ADDITIONAL MATERIAL

A model-based optimization framework for the inference of regulatory interactions using time-course DNA microarray expression data

Reuben Thomas^1^, Carlos J. Paredes ^4,5^, Sanjay Mehrotra^2^, Vassily Hatzimanikatis^3,*^

and Eleftherios T. Papoutsakis^4,6*^,

^1^Laboratory of Molecular Toxicology, National Institute of Environmental Health Sciences, National Institutes of Health, Research Triangle Park, North Carolina, USA; ^2^Department of Industrial Engineering and Management Science, Northwestern University, Evanston, Illinois 60208-3120, USA; ^3^Laboratory of Computational Systems Biotechnology, EPFL, CH-1015 Lausanne, Switzerland; and ^4^Department of Chemical and Biological Engineering, Northwestern University, Evanston, Illinois 60208-3120, USA; ^5^ Current address: Gevo, Inc., 133 N. Altadena Dr. Suite 310, Pasadena, CA  91107, USA. ^6^ Current address: Dept. of Chemical Engineering and the Delaware Biotechnology Institute, University of Delaware, Newark, DE 19711.

Email: Reuben Thomas – [thomasr3@niehs.nih.gov](mailto:thomasr3@niehs.nih.gov); Carlos J. Paredes – [c-paredes@northwestern.edu](mailto:c-paredes@northwestern.edu); Sanjay Mehrotra – [mehrotra@iems.northwestern.edu](mailto:mehrotra@iems.northwestern.edu), Vassily Hatzimanikatis – [vassily.hatzimanikatis@epfl.ch](../vassily.hatzimanikatis@epfl.ch); Eleftherios T. Papoutsakis – [e-paps@northwestern.edu](mailto:e-paps@northwestern.edu)

^*^Corresponding authors

Contact Information:

| Vassily Hatzimanikatis  Laboratory of Computational Systems Biotechnology,  EPFL, CH-1015 Lausanne, Switzerland  Email: vassily.hatzimanikatis@epfl.ch  Tel: +41 (0)21 693 98 70  Fax: +41 (0)21 693 98 75 | Eleftherios T. Papoutsakis,  Department of Chemical and Biological Engineering, Northwestern University, Evanston, Illinois 60208-3120, USA.  Email: [e-paps@northwestern.edu](mailto:e-paps@northwestern.edu)  Tel: 001-847-491-7455  Fax: 001-847-491-3728 |
| --- | --- |

Keywords: Gene Regulation, S-systems, DNA arrays, Time-varying, Optimization, *Bacillus anthracis*

Running Title: “Performance of model-based methods for network inference”

# A1. Dynamic characteristics of the transcription/translation model

In this section we analyze the way the parameters of the S-system based regulation model (Equation 1, Main paper) affect the dynamic characteristics of the different mRNAs and proteins in the system. The dynamic characteristics considered include,

1. The stability of the system around a steady state.
2. The time necessary to reach steady state.
3. The collinearity or the near-collinearity between the time profiles of the different mRNA and protein concentrations.
4. Sensitivity of the dynamic behavior to the parameters of the model.

In order to analyze these four issues, the required analysis for the general model framework is very complex. If however, we make the assumption that the rates of synthesis of any of the mRNAs and proteins are never more than, for example, twice their corresponding degradation rates, then the required analysis becomes easy. At steady state, the rates of synthesis and degradation are equal. Hence, intuitively, we are trying to analyze the evolution of the system from a time point that is not too ‘far away’ from the steady state. Mathematically at any time *t*,

$$0\leq\frac{V_{sm,i}}{V_{dm,i}}\leq2 , 0\leq\frac{V_{sp,i}}{V_{dp,i}}\leq2$$

for the *i*^th^ mRNA and protein,

$\begin{matrix} \Rightarrow-1\leq\left(\frac{V_{sm,i}}{V_{dm,i}}-1\right)\leq1 , -1\leq\left(\frac{V_{sp,i}}{V_{dp,i}}-1\right)\leq1 \\ \Rightarrow\left|\left(\frac{V_{sm,i}}{V_{dm,i}}-1\right)\right|\leq1, \left|\left(\frac{V_{sp,i}}{V_{dp,i}}-1\right)\right|\leq1 \end{matrix}$ (A.1)

From Equation (1) in the main paper,

$$\begin{matrix} \frac{1}{V_{dm,i}}\frac{dm_{i}}{dt}=\frac{V_{sm,i}}{V_{dm,i}}-1, \frac{1}{V_{dp,i}}\frac{dp_{i}}{dt}=\frac{V_{sp,i}}{V_{dp,i}}-1 \\ \Rightarrow\frac{1}{\beta_{i}m_{i}}\frac{dm_{i}}{dt}=\frac{V_{sm,i}}{V_{dm,i}}-1, \frac{1}{\delta_{i}p_{i}}\frac{dp_{i}}{dt}=\frac{V_{sp,i}}{V_{dp,i}}-1 \\ \Rightarrow\frac{1}{\beta_{i}}\frac{d\log(m_{i})}{dt}=\frac{V_{sm,i}}{V_{dm,i}}-1, \frac{1}{\delta_{i}}\frac{d\log(p_{i})}{dt}=\frac{V_{sp,i}}{V_{dp,i}}-1 \end{matrix}$$

Therefore Equation A.1 implies

$\left|\frac{1}{\beta_{i}}\frac{d\log(m_{i})}{dt}\right|\leq1, \left|\frac{1}{\delta_{i}}\frac{d\log(p_{i})}{dt}\right|\leq1$ (A.2)

This condition will convert the non-linear system of differential equations (Equation (1), main paper) to a linear system of differential equations in the logarithmic space. The steps to achieve this goal are given below.

From the mass balance requirement of the *i*^th^ protein,

$$\begin{matrix} \frac{1}{\delta_{i}p_{i}}\frac{dp_{i}}{dt}=\frac{\gamma_{i}m_{i}}{\delta_{i}p_{i}}-1 \Rightarrow1+\frac{1}{\delta_{i}}\frac{d\log(p_{i})}{dt}=\frac{\gamma_{i}m_{i}}{\delta_{i}p_{i}} \\ \log(m_{i})=\log(p_{i})+\log\left( 1+\frac{1}{\delta_{i}}\frac{d\log(p_{i})}{dt}\right)+\log\left(\frac{\delta_{i}}{\gamma_{i}}\right) \end{matrix}$$

Using Equation A.2 and the fact that,

$\log(1+x)\cong x$ when $\left|x\right|\leq1$ (A.3)

$\begin{matrix} \log(m_{i})\cong\log(p_{i})+ \frac{1}{\delta_{i}}\frac{d\log(p_{i})}{dt}+\log\left(\frac{\delta_{i}}{\gamma_{i}}\right) \\ \Rightarrow\frac{d\log(m_{i})}{dt}\cong\frac{d\log(p_{i})}{dt}+\frac{1}{\delta_{i}}\frac{d^{2}\log(p_{i})}{dt^{2}} \end{matrix}$ (A.4)

The mass balance requirement of the *i*^th^ mRNA implies,

$$\frac{1}{\beta_{i}m_{i}}\frac{dm_{i}}{dt}=\frac{\alpha_{i}\prod_{j=1}^{n} p_{j}^{\varepsilon_{ij}}}{\beta_{i}m_{i}}-1 \Rightarrow\log\left(1+\frac{1}{\beta_{i}}\frac{d\log(m_{i})}{dt}\right)+\log\left(\frac{\beta_{i}}{\alpha_{i}}\right)=\sum_{j=1}^{n} \varepsilon_{ij}\log(p_{j})-\log(m_{i})$$

Using Equation A.3,

$$\frac{1}{\beta_{i}}\frac{d\log(m_{i})}{dt}+\log\left(\frac{\beta_{i}}{\alpha_{i}}\right)\cong\sum_{j=1}^{n} \varepsilon_{ij}\log(p_{j})-\log(m_{i})$$

Using Equations A.3 and A.4,

(A.5)

where,

Now, we introduce the following definitions:

- A *n*×*n* matrix *E* whose (*i*,*j*)^th^  element is given by $\beta_{i}\delta_{i}\bar{\varepsilon}_{ij}$.
- A 2*n*×1 vector *b* whose first *n* components are zero and for the remaining *n* components the (*n*+*i*)^th^ element is given by $\beta_{i}\delta_{i}\log\left(\frac{\beta_{i}\delta_{i}}{\alpha_{i}\gamma_{i}}\right)$.
- An *n*×1 vector *b^s^* whose *i*^th^ component is given by $\beta_{i}\delta_{i}\log\left(\frac{\beta_{i}\delta_{i}}{\alpha_{i}\gamma_{i}}\right)$.
- A 2*n*×1 vector *x* whose first *n* components the logarithm of *n* protein concentrations and the next *n* components are the first derivatives (with respect to time) of the log of the *n* protein concentrations at a given time *t*.
- *I*, the *n*×*n* identity matrix.
- *O*, be the n×*n* zero matrix.
- D a *n*×*n* diagonal matrix whose *i*^th^ diagonal component is given by $-(\beta_{i}+\delta_{i})$.
- A 2*n*×2*n* matrix A given by,

$$A=\left[\begin{matrix} O & I \\ E & D \end{matrix}\right]$$

Using the above definitions Equation (A.5) for all the *i* genes can be written in a compact matrix-vector notation as a set of linear differential equations,

$\frac{dx}{dt}=Ax-b$ (A.6)

At steady state, the protein concentrations do not change with time, i.e., their derivatives are zero. The log of the protein concentrations at steady state are given by a solution to the system of equations,

$Ey=b^{s}$ (A.7)

Note that the stability of the system requires that the above system of equations does have at least one solution or *b^s^* lies in the range space of the matrix operator *E*, i.e.,

$$b^{s}\in Range(E)$$

Now, we derive the solution of Equation (A.6) in terms of the eigenvalues and eigenvectors of the matrix *A*. Unless the parameters $\beta_{i}, \delta_{i}, \varepsilon_{ij }$are related in a definite manner (e.g. all or some of them are exactly equal), matrix *A* will not have repeated eigenvalues. Hence *A* will have 2*n* distinct (possibly complex) eigenvalues. Let the eigenvalues of *A* be given by, $\left\{\lambda_{i}\right\}_{i=1}^{2n}$ and the corresponding set of eigenvectors be given by $\left\{v_{i}\right\}_{i=1}^{2n}$. Then

$$x(t)=\sum_{j=1}^{2n} c_{i}e^{\lambda_{i}t}v_{i}+v_{p}$$

where $v_{p}$is a solution of Equation (A.7). $c_{i}$s are constants whose values are determined by the initial conditions.

A1.1 Stability of the system

For stability the real part of the all the eigenvalues should be negative. Therefore, for the stability of the system two conditions need to be met,

$\begin{matrix} b^{s}\in Range(E) \\ \mathrm{Re}(\lambda_{i})<0, i=1,2,\ldots,n \end{matrix}$ (A.8)

A1.2 Collinearity between time profiles

Let $\mu(t)$denote a 2*n*×1 vector whose *i*^th^ component is given by $c_{i}e^{\lambda_{i}t}$, *X* denote a 2*n*×*N_s_* matrix whose column vectors are the vector *x* at *N_s_* distinct time points, *Q* be the 2*n*×2*n* matrix whose column vectors are the eigenvectors of the matrix *A*. $\Lambda$a 2*n*× *N_s_* matrix whose column vectors denote are the vector $\mu$at the *N_s_* time points. Then,

$X=Q\Lambda$ (A.9)

Then similarity between the time profiles of the different proteins is associated with the conditioning of the matrix *X*. The conditioning of *X* in turn depends on the conditioning of the matrices *Q* and $\Lambda$. If the condition number of *X* is denoted by cond(*X*) then the following relation holds true ([1])

$$cond(X)\leq cond(Q)\cdot cond(\Lambda)$$

The condition number of the matrix $\Lambda$would be high if all or some of the eigenvalues are close to each other. It would also be high if, over the chosen *N_s_* time points, the vector $\mu$ does not change much, i.e., the system evolves at a slow rate. This would happen in the scenario when the real part of the different eigenvalues is close to zero and the *N_s_* samples are chosen only for a relatively small time period.

The condition number of the matrix *Q* would be high if the all or some of its row vectors are close to being linearly dependent. An example of a situation when this occurs is the following. Suppose the network of interactions between the *n* genes in the system can be split into two disjoint sub-networks of interactions, each with *n*/2 genes. Also suppose that the two sub-networks are exactly similar in structure and parameter values, i.e., there is a one-to-one correspondence between the genes in the two sub-networks. Then, corresponding genes in the two sub-networks would behave in the exact same manner even though they don’t interact with each other. A detailed analysis of how the parameters and the structure of the network of interactions between the genes affect the linear dependencies between genes is left as future work.

A1.3 Sensitivity of the dynamic behavior to the parameters of the model

The method proposed in this paper requires estimates of the half-lives of the different mRNAs and proteins in the system. The issue here is how accurate do these estimates have to be in order that for the dynamic behavior to be not too different from what it really is. We have just seen that the dynamic behavior is governed by the eigenvalues and eigenvectors of the matrix *A*. So the thing to do would be to check how sensitive the eigenvalues and eigenvectors of *A* are to errors in its elements. Using a result from Horn and Johnson [1], we can claim that the perturbation of the eigenvalues of *A* due to errors in its elements depends on the condition number of the eigenvector matrix, *Q*. Let *Z* be a matrix representing the errors in the components of *A*. Let $\hat{\lambda}$be an eigenvalue of *A*+*Z*. There exists an eigenvalue of *A*, $\lambda_{j}$such that,

where || || denotes the matrix norm.

A2 Error in prediction of protein concentrations

Let $M_{i}(t)$and $P_{i}(t)$be the true mRNA and protein concentrations as a function of time *t*, from time 0 to time *T* and let $M_{i}^{a}(t)$and $P_{i}^{a}(t)$denote their approximations. The mRNA

concentrations are approximated as splines while the protein concentrations are obtained by using the mRNA approximations in the protein mass-balance equations. At any time *t*,

$$\begin{matrix} \frac{dP_{i}(t)}{dt}=\gamma_{i}M_{i}(t)-\delta_{i}P_{i}(t) \\ \frac{dP_{i}^{a}(t)}{dt}=\gamma_{i}M_{i}^{a}(t)-\delta_{i}P_{i}^{a}(t) \end{matrix}$$

Therefore,

$$\begin{matrix} \frac{d(P_{i}(t)-P_{i}^{a}(t))}{dt}+\delta_{i}(P_{i}(t)-P_{i}^{a}(t))=\gamma_{i}(M_{i}(t)-M_{i}^{a}(t))\approx O(\gamma_{i}TN_{S}^{-4}) \\ \Rightarrow(P_{i}(t)-P_{i}^{a}(t))\approx O(\gamma_{i}TN_{S}^{-4})+(P_{i}(0)-P_{i}^{a}(0))e^{-\delta t} \end{matrix}$$

Hence the error in approximating the protein concentrations is a function of the error in approximating in the mRNA concentrations and the error in the estimation of the initial protein concentration. Notice that as time increases the error due to the estimation of initial protein concentration decreases.

A3. Data for the synthetic networks

Define the interactions in the network (the elasticities, *ε*_ij_) by the matrix *G*. The notation of the other parameters is the same as used in the main paper.

A3.1 “Low” network

Table A.1: Interactions corresponding to the genes from the “Low” network whose time-course profiles are characterized by a relatively low degree of similarity. The strength of all interactions is 0.5. A positive sign corresponds to an activating interaction, a negative sign to an inhibiting interaction and a zero value to an absent interaction. A row corresponds to a regulated gene and a column corresponds to a regulator gene.

|  | 1 | 2 | 3 | 4 | 5 | 6 | 7 | 8 | 9 | 10 |
| --- | --- | --- | --- | --- | --- | --- | --- | --- | --- | --- |
| 1 | -0.5 | -0.5 | 0 | 0 | 0.5 | 0 | 0 | 0 | 0 | 0 |
| 2 | 0 | 0 | 0 | -0.5 | 0 | -0.5 | 0 | 0 | -0.5 | 0 |
| 3 | -0.5 | 0.5 | 0 | 0 | 0 | 0 | 0 | 0 | 0 | -0.5 |
| 4 | 0.5 | 0 | 0 | 0 | 0 | 0 | -0.5 | 0 | 0 | 0.5 |
| 5 | 0 | 0 | 0 | 0.5 | 0 | 0 | 0 | -0.5 | 0 | -0.5 |
| 6 | 0 | 0.5 | 0 | 0 | 0 | 0.5 | 0.5 | 0 | 0 | 0 |
| 7 | 0 | 0 | -0.5 | 0 | 0.5 | -0.5 | 0 | 0 | 0 | 0 |
| 8 | 0 | 0 | 0 | -0.5 | 0 | -0.5 | 0 | -0.5 | 0 | 0 |
| 9 | 0 | 0.5 | 0 | 0 | 0 | 0 | -0.5 | -0.5 | 0 | 0 |
| 10 | 0 | 0 | 0 | 0 | 0 | -0.5 | 0 | 0 | -0.5 | -0.5 |

Table A.2: The values of the different parameters for the 10 genes of the “Low” network whose time-course profiles are characterized by a relatively low degree of similarity. *m*^0^ and *p*^0^ are given in concentration units. β and δ are given in inverse time units while α and γ are in units that are consistent with the utilized units of concentration and time.

|  | α | β | γ | δ | *m*^0^ | *p*^0^ |
| --- | --- | --- | --- | --- | --- | --- |
| 1 | 0.25 | 0.59 | 0.22 | 0.38 | 1.00 | 2.00 |
| 2 | 0.20 | 0.23 | 0.11 | 0.14 | 3.00 | 4.00 |
| 3 | 0.28 | 0.40 | 0.27 | 0.35 | 5.00 | 6.00 |
| 4 | 0.70 | 0.05 | 0.30 | 0.29 | 7.00 | 8.00 |
| 5 | 0.71 | 0.52 | 0.24 | 0.31 | 9.00 | 10.00 |
| 6 | 0.06 | 0.35 | 0.18 | 0.30 | 11.00 | 12.00 |
| 7 | 0.16 | 0.65 | 0.24 | 0.11 | 13.00 | 14.00 |
| 8 | 0.11 | 0.22 | 0.11 | 0.21 | 15.00 | 16.00 |
| 9 | 0.00 | 0.59 | 0.15 | 0.04 | 17.00 | 18.00 |
| 10 | 0.70 | 0.69 | 0.08 | 0.29 | 19.00 | 20.00 |

A3.2 “Medium” network

Table A.3: Interactions corresponding to the genes from the “Medium” network whose time-course profiles are characterized by a relatively medium degree of similarity. The strength of all interactions is 0.5. A positive sign corresponds to an activating interaction, a negative sign to an inhibiting interaction and a zero value to an absent interaction. A row corresponds to a regulated gene and a column corresponds to a regulator gene.

|  | 1 | 2 | 3 | 4 | 5 | 6 | 7 | 8 | 9 | 10 |
| --- | --- | --- | --- | --- | --- | --- | --- | --- | --- | --- |
| 1 | -0.5 | 0 | 0 | 0 | 0 | 0 | 0 | -0.5 | -0.5 | 0 |
| 2 | 0.5 | -0.5 | 0 | 0 | 0 | 0 | 0 | -0.5 | 0 | 0 |
| 3 | 0.5 | 0.5 | 0 | 0 | 0.5 | 0 | 0 | 0 | 0 | 0 |
| 4 | 0 | 0.5 | 0 | -0.5 | 0 | -0.5 | 0 | 0 | 0 | 0 |
| 5 | 0 | 0 | 0 | -0.5 | -0.5 | 0 | 0 | 0 | 0 | -0.5 |
| 6 | 0 | 0 | 0.5 | 0 | 0 | 0 | -0.5 | 0 | 0 | -0.5 |
| 7 | 0 | 0 | -0.5 | 0 | 0 | 0 | 0 | 0.5 | 0 | -0.5 |
| 8 | 0 | 0 | 0.5 | 0 | 0 | 0 | -0.5 | 0 | 0 | -0.5 |
| 9 | 0 | 0 | -0.5 | 0 | 0 | 0.5 | 0 | 0.5 | 0 | 0 |
| 10 | 0 | -0.5 | 0 | 0 | -0.5 | 0.5 | 0 | 0 | 0 | 0 |

Table A.4: The values of the different parameters for the 10 genes of the “Medium” network whose time-course profiles are characterized by a relatively medium degree of similarity. *m*^0^ and *p*^0^ are given in concentration units. β and δ are given in inverse time units while α and γ are in units that are consistent with the utilized units of concentration and time.

|  | α | β | γ | δ | *m*^0^ | *p*^0^ |
| --- | --- | --- | --- | --- | --- | --- |
| 1 | 0.61 | 0.31 | 0.09 | 0.03 | 1.00 | 2.00 |
| 2 | 0.57 | 0.66 | 0.04 | 0.01 | 3.00 | 4.00 |
| 3 | 0.44 | 0.03 | 0.02 | 0.02 | 5.00 | 6.00 |
| 4 | 0.56 | 0.06 | 0.02 | 0.01 | 7.00 | 8.00 |
| 5 | 0.51 | 0.12 | 0.05 | 0.10 | 9.00 | 10.00 |
| 6 | 0.22 | 0.73 | 0.07 | 0.07 | 11.00 | 12.00 |
| 7 | 0.24 | 0.51 | 0.02 | 0.07 | 13.00 | 14.00 |
| 8 | 0.07 | 0.36 | 0.07 | 0.02 | 15.00 | 16.00 |
| 9 | 0.27 | 0.58 | 0.09 | 0.04 | 17.00 | 18.00 |
| 10 | 0.10 | 0.09 | 0.07 | 0.08 | 19.00 | 20.00 |

A3.3 “High” network

Table A.5: Interactions corresponding to the genes from the “High” network whose time-course profiles are characterized by a relatively high degree of similarity. The strength of all interactions is 0.5. A positive sign corresponds to an activating interaction, a negative sign to an inhibiting interaction and a zero value to an absent interaction. A row corresponds to a regulated gene and a column corresponds to a regulator gene.

|  | 1 | 2 | 3 | 4 | 5 | 6 | 7 | 8 | 9 | 10 |
| --- | --- | --- | --- | --- | --- | --- | --- | --- | --- | --- |
| 1 | 0 | -0.5 | 0 | -0.5 | 0 | 0 | 0 | 0 | 0 | -0.5 |
| 2 | 0 | 0 | -0.5 | 0 | 0 | 0 | 0 | 0.5 | 0 | 0.5 |
| 3 | 0.5 | 0.5 | 0.5 | 0 | 0 | 0 | 0 | 0 | 0 | 0 |
| 4 | 0 | 0 | 0 | 0 | 0 | 0 | 0 | 0.5 | -0.5 | 0.5 |
| 5 | 0 | 0 | 0 | 0 | 0 | 0 | -0.5 | 0.5 | -0.5 | 0 |
| 6 | 0 | 0 | 0 | 0 | 0 | -0.5 | 0.5 | 0 | 0 | -0.5 |
| 7 | 0.5 | 0 | 0 | 0 | 0 | 0 | -0.5 | -0.5 | 0 | 0 |
| 8 | 0 | -0.5 | 0 | -0.5 | -0.5 | 0 | 0 | 0 | 0 | 0 |
| 9 | -0.5 | 0 | 0 | 0 | 0 | 0.5 | 0 | 0 | -0.5 | 0 |
| 10 | 0.5 | 0 | 0 | 0 | 0 | -0.5 | 0 | -0.5 | 0 | 0 |

Table A.6: The values of the different parameters for the 10 genes of the “High” network whose time-course profiles are characterized by a relatively high degree of similarity. *m*^0^ and *p*^0^ are given in concentration units. β and δ are given in inverse time units while α and γ are in units that are consistent with the utilized units of concentration and time.

|  | α | β | γ | δ | *m*^0^ | *p*^0^ |
| --- | --- | --- | --- | --- | --- | --- |
| 1 | 0.071 | 0.080 | 0.017 | 0.007 | 1.00 | 2.00 |
| 2 | 0.024 | 0.036 | 0.001 | 0.000 | 3.00 | 4.00 |
| 3 | 0.080 | 0.005 | 0.015 | 0.008 | 5.00 | 6.00 |
| 4 | 0.081 | 0.030 | 0.016 | 0.004 | 7.00 | 8.00 |
| 5 | 0.062 | 0.010 | 0.011 | 0.004 | 9.00 | 10.00 |
| 6 | 0.066 | 0.014 | 0.007 | 0.010 | 11.00 | 12.00 |
| 7 | 0.048 | 0.017 | 0.014 | 0.004 | 13.00 | 14.00 |
| 8 | 0.093 | 0.066 | 0.017 | 0.008 | 15.00 | 16.00 |
| 9 | 0.042 | 0.043 | 0.005 | 0.003 | 17.00 | 18.00 |
| 10 | 0.003 | 0.011 | 0.004 | 0.008 | 19.00 | 20.00 |

A4 Issues affecting the performance of the proposed method

A4.1 Data for missing genes

Table A.7: Indices of the genes that was present in the different experiments involving missing genes on the “Low” network whose time-course profiles are characterized by a relatively low degree of similarity.

| Set no. | Genes in the set | Fraction of correctly identified interactions |
| --- | --- | --- |
| 1 | {1,3,4,5,6,7, 8,9,10} | 22/23 |
| 2 | {2,3,4,5,6,7, 8,9,10} | 22/25 |
| 3 | {1,3,4,9,10, 7,8,2} | 9/18 |
| 4 | {1,3,4,5,6,7, 9,10} | 16/18 |
| 5 | {1,3,4,5,6,7, 9} | 7/14 |
| 6 | {1,2,3,4,5,7,9} | 10/14 |
| 7 | {1,3,4,5,7,8} | 5/11 |
| 8 | {3,4,5,6,7,9} | 5/8 |
| 9 | {2,6,8,9,10} | 9/11 |
| 10 | {1,3,4,5,7} | 4/8 |

A4.2 Number of iterations of heuristic method


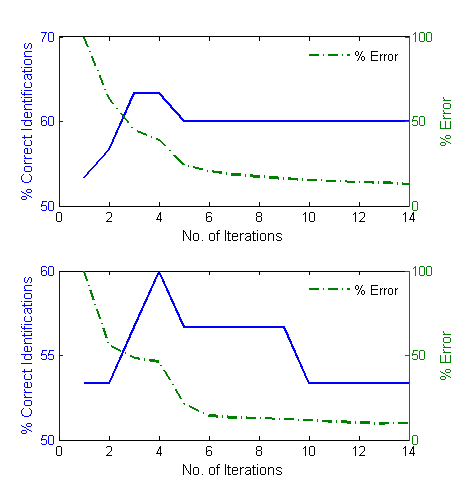


Figure A.1: Variation of the percentage of correctly identified interactions among 30 known interactions and the error as a percentage of the error obtained at the first iteration. The variation is with respect to the number of iterations of the Coordinate descent heuristic method. The two plots indicate the progress from two different starting points for the heuristic method. The “experimental” data is obtained from simulations using the “Low” synthetic network (Figure 1). The two plots indicate the progress from two different starting points.

A4.3 Effect of error in the estimates of half-lives of mRNAs and proteins

The effect of errors in the estimates of the half-lives of the mRNAs and proteins in the system was tested using the data from the “Low” synthetic network. Different cases were considered where the half-lives are known only within a certain percentage of the true values. For each case, 50 samples of expression data were generated using these noisy estimates of half-lives. The inference of the connections was done using these data. Like it was done elsewhere in the paper, we assumed the parameters $\gamma_{i},m_{i}^{c},p_{i}(0)$ to be known to avoid basing our conclusions on local solutions. The results are shown in Table A.8. We observe that the percentage correct identifications are relatively robust to errors in the estimates of the half-lives. Only when the half-lives are distributed within a 100% interval around its true values do we see the percentage correct identifications approaching that of a random method (See Section 2.6.1).

**Table A.8**: Variation of the percentage of correctly identified interactions (given at a 95% confidence interval) with the percentage error in the estimates of half-lives of all the mRNAs and proteins. E.g., a 5% error implies that all the half-life data are known only with an error of ±5% of the true values. The half-life parameters from the “Low” synthetic network were used here.

| %Error | %Correct |
| --- | --- |
| 0.1 | 78.1±0.8 |
| 1 | 65.9±1.5 |
| 5 | 60.5±1.2 |
| 10 | 57.3±1.7 |
| 20 | 51.0±1.8 |
| 50 | 34.5±3.0 |
| 100 | 17.7±2.5 |

A5. *Bacillus anthracis* data retrieval from literature source

A5.1 Data retrieval

One of the most complex yet well-studied regulatory networks is the sporulation cascade in *B. subtilis*. With minor variations this network seems to be well preserved in bacillus and clostridia organisms ([2]). Liu *et al.* [3] performed DNA array experiments on the set of genes involved in the formation of the *B. anthracis* endospore. Using the array data, the authors identified five clusters of genes representing different stages involved in development of the endospore over time. We retrieved the data that the authors deposited on the GEO database ([4]) with reference GSE840 and normalized and averaged the slides using a segmental nearest neighbor logarithmic expression ratio-of-the mean (SNN-LERM) approach ([5], [6]) coded in MATLAB ([7]).

A subset of the genes depicted in Figure A.2 were chosen as they play pivotal roles on the sporulation cascade and their interactions are documented through the presence of either binding sites or mutant strains ([8]-[11]).


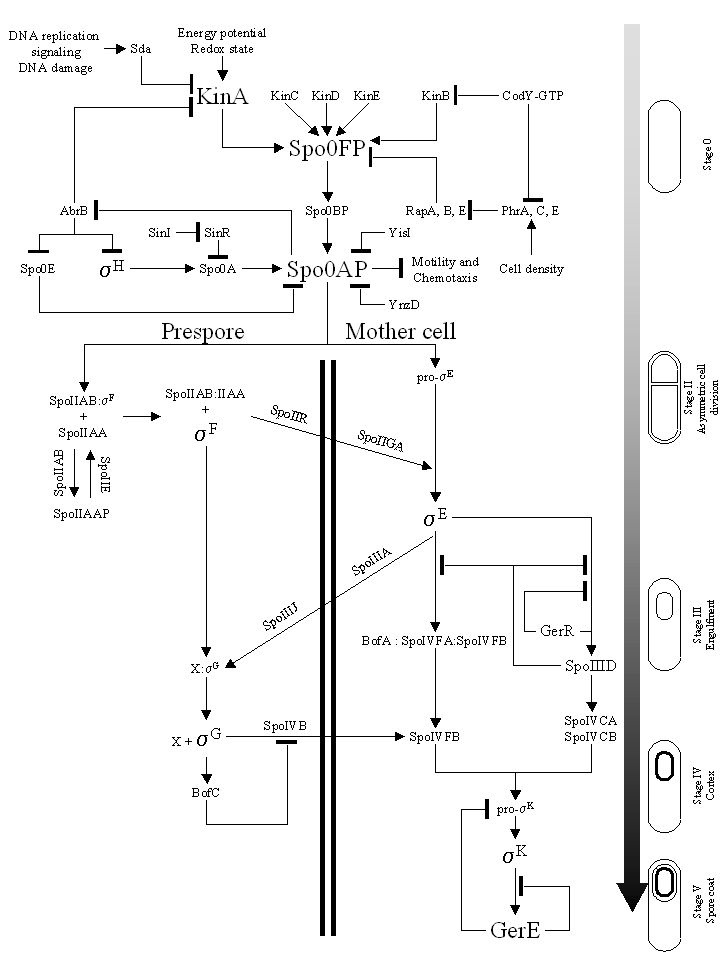


Figure A.2: Subset of genes involved in the sporulation cascade on *B. anthracis*. Pointed arrows indicate activating interactions while flat-headed arrows indicate inhibiting interactions between genes at the two ends of the arrows.

**A5.2 Choice of the 9-gene subset**

This section elaborates on the biological basis for the choice of the 9-gene subset in *B. anthracis* that is used in the paper. The discussion below is based on the general reference for *B. subtilis*, the well-studied organism similar to *B. anthracis*, by Sonenshein *et al*. [8].

Spo0F is used here as the starting point of the network as the profile for kinA (main sporulation sensor kinase) was incomplete ([3]). As discussed in the main paper, its profile also serves as a replacement of that of *spo0A* and in particular of the activated Spo0A, namely Spo0A~P.

SigF is the first sigma factor in the pre-spore cell compartment and its role is crucial in spore development, so it must be included in the chosen network.

SigE is the first sigma factor on the mother cell, is a crucial one for the sporulation cascade, and thus must be included in the network. Although its transcription is Spo0A~P dependent, the active SigE protein requires processing by sporulation proteins which are controlled by SigF.

SpoIIIJ null mutations arrest sporulation by blocking SigG activity, and is retained in the network solely to reflect this fact. *spoIIIJ* is transcribed during vegetative growth from an atypical SigA promoter and is shut down around the early stationary phase in *B. subtilis*.

SigG is the second transcription factor in the prespore cell compartment, and is retained in the network based on its biological importance.

SpoIIID stimulates the transcription from some SigE and SigK-dependent promoters, while repressing transcription from some others. Its transcription occurs in the mother cell from a SigE promoter activated by SpoIIID itself. It plays a crucial role in sporulation and the data show a rather distinct profile, thus the decision to include it in the network.

According to the available literature, transcription of SpoIVFB starts in the mother cell from a SigE promoter. It plays a crucial role in sporulation and the data show a rather distinct profile, thus the decision to include it in the network.

SigK is the last of the major sigma factors of sporulation necessary for the expression of the genes/proteins for spore morphogenesis. The most characteristic gene of its regulon is *gerE*. These two gene have rather distinct expression patterns, and are thus included in the network.

**A6. *Bacillus anthracis* data input for the inference algorithm**

The inputs to the inference algorithm were the smoothing splines and the bounds of the different variables involved in the optimization.

## A6.1. Smoothing splines

Smoothing splines were fit to the expression ratio time profiles of the chosen set of 9 genes. Smoothing parameters were determined using either the Generalized Cross Validation (GCV) technique ([12]) or by trial and error where the curves (determined by the GCV technique) did not seem to capture the peaks in the expression ratios at the appropriate time points. The figure below gives the plots of the different expression ratios with time that include the experimental measurements and the smoothing splines.


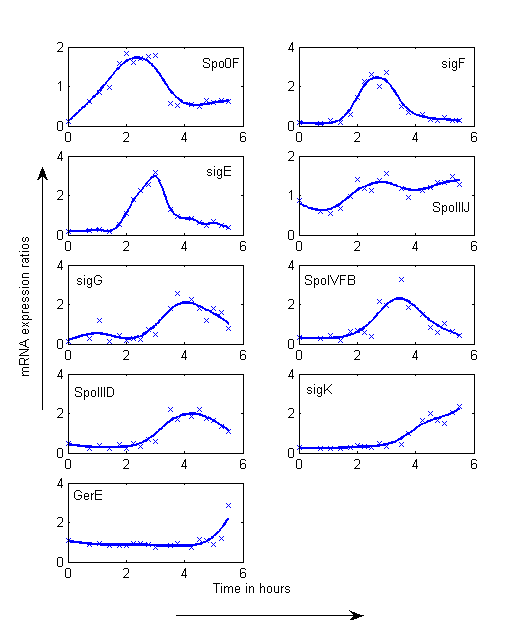


**Figure A.3:** Time profiles of the expression ratios of the chosen subset of genes from the sporulation cascade of *B. anthracis*. The continuous curves are the smoothing splines obtained from the experimental data indicated by ‘x’ on the graphs

## A6.2 Units and bounds on variables and values of half-lifes of mRNAs and proteins

The half-lives of the mRNAs and proteins are used to compute the values of the parameters, β and δ. The half-lives of the different mRNAs were kindly provided by Prof. L. Hederstedt ([13]) are given in Table A.8. These half-lives are used to compute the parameters (β). The half-lives of *B. anthracis* proteins were estimated by using the N-end rule ([14]). They were estimated to be between 3-7 hours. Therefore the half-lives of the proteins were chosen randomly from this range and provided in Table A.8.

**Table A.8:** Half-lives of the different mRNAs and proteins corresponding to the chosen subset of genes from the sporulation cascade of *B. anthracis.* Data are from a similar organism, *B. subtilis* ([13], [14])*.*

| Gene | mRNA half-life (minutes) | protein half-life (hours) |
| --- | --- | --- |
| spo0F | 1.00 | 4.03 |
| *sigF* | 6.00 | 3.14 |
| *sigE* | 2.00 | 3.58 |
| *spoIIIJ* | 4.00 | 4.17 |
| *sigG* | 3.00 | 4.01 |
| *spoIVFB* | 4.00 | 3.61 |
| *spoIIID* | 2.00 | 3.86 |
| *sigK* | 4.00 | 3.23 |
| *gerE* | 12.00 | 3.37 |

The different variables involved are the elasticities (ε), the chemical kinetic constants (α and γ), the experimental reference states (*m*^c^) and the initial protein concentrations (*P*^0^). Typical values of the elasticities under different kinetic mechanisms like Michaelis-Menten and Hill kinetics are less than one ([15]). The absolute value of these variables control the steepness of the time profile curves – larger the values the steeper the curves could be. Taking these facts into consideration, a bound of 2 was imposed on the absolute values of elasticities.

Gene expressions were assumed to be in the units of mRNA copies per cell and proteins in protein copies per cell. We know that at any time the number of copies of mRNA per cell ranges from 0-10 while the number of copies of proteins ranges from 0-1000. Most of the genes were expressed only for a relatively short period of time over the course of the experiment. As described in the main paper, the reference state (*m*^c^) can be interpreted as the average of the number of mRNA copies per cell over the time course of the experiment. Therefore, a bound of 1 copy/cell was applied to the variables corresponding to the reference states. A bound of 1000 copies/cell was applied to the variables corresponding to the initial values of the protein concentrations (*P^0^*).

We now present back-of-the-envelope calculations for bounds on the parameters, α and γ. To derive the bounds for the variables corresponding to the translation rates (γ), we try to get bounds for the synthesis rates of the proteins. Getting bounds on the degradation rates of the different proteins is easy because we have knowledge of the half-lives of proteins (24 minutes–7 hours) and bounds on their concentrations (1000 copies/cell). The degradation rates of the proteins are then used as an indicator of the synthesis rates. If a protein is present then we assume that there is at least one copy of the corresponding mRNA per cell (to allow for the synthesis of the protein). Then, using bounds on the number of copies of mRNA per cell (1-10 copies/cell) and the protein mass-balance equation (Equation1, main paper), we can thus derive bounds for γ.

$$\begin{matrix} V_{dp}\leq\frac{\log(2)}{0.4}*1000 \text{hr}^{\text{-1}}\cong1800 \text{hr}^{\text{-1}} \\ \Rightarrow\gamma\cdot m\leq1800 \text{hr}^{\text{-1}} \Rightarrow\gamma\leq1800 \text{hr}^{\text{-1}} \end{matrix}$$

Since we bound all other variables and parameters in the system the values of the parameter α are implicitly bounded.

Therefore in summary,

$$\begin{matrix} \alpha\geq0 \\ 0\leq\gamma\leq1800 \text{hr}^{\text{-1}} \\ 0\leq p^{0}\leq1000 \text{copies}/\text{cell} \\ 0\leq m^{c}\leq1 \text{copy}/\text{cell} \\ -2\leq\varepsilon\leq2 \end{matrix}$$

A7. Further biological discussion of the results of Table 4

The discussion below is again based on the general reference for *B. subtilis*, the well-studied organism similar to *B. anthracis*, by Sonenshein *et al.* [8].

The inferred regulation of *spo0F* (activation by *gerE* and repression by *spoIIID*) seems to be biologically not sound. Based on the profiles of *gerE* and *spo0F*, it seems that the *spo0F* activation by *gerE* is a numerical artifact that appears to help minimize the error between the observed and calculated profile of *spo0F*. The role of *spoIIID* as repressor of *spo0F* does not have any bibliographical support. However, none of putative *B. subtilis* repressors of *spo0F* (RapA, B, E) has been described in *B. anthracis,* so the computed role of *spoIIID* as a repressor indicates that the decay of *spo0F* mRNA is too fast to be attributed only to the half life of its mRNA and might require repression by a gene (or genes) with a transcriptional profile similar to that of *spoIIID*.

The transcription of *sigF* is Spo0A~P dependent, so its activation by *spo0F*/Spo0A~P reflects this fact. It is not known which genes (if any) repress the transcription of *sigF*, so its repression by SpoIIID can be interpreted along the same lines than that of *spo0F* as discussed above.

Again, it is not known which genes (if any) repress the transcription of *sigE*, so its repression by SpoIIID can be interpreted along the same lines as those in the case of *spo0F* and *sigF* above.

Regarding *spoIIIJ*, the lack of further information about its regulation makes difficult to explain its profile in *B. anthracis*, and its putative regulation by an early stage sporulation protein, here computed to be Spo0F (Table 4) cannot be ruled out.

Transcription of *sigG* takes place from a SigF promoter although its transcription also requires the presence of an active SigE. The interaction between SigE and SigG seems to take place through SpoIIIA and SpoIIIJ. As the transcriptional profile of spoIIIJ does not seem to convey much information regarding its timing, it seems logical to assume that the profile of SigE could be a good compromise (albeit somehow early) as a positive regulator of *sigG*. The downregulation of *sigG* by *spo0F* does not seem to have any biological meaning. We attribute this interaction as a requirement of the numerical solution to keep the transcriptional profile of *sigG* very low during the early part of sporulation when SigE is active but SigG is inactive.

As discussed above, the transcription of *spoIIID* occurs in the mother cell from a SigE promoter activated by SpoIIID itself. Thus, the dependence of *spoIIID* expression from SigE has been correctly captured in the results (Table 4), however the calculations do not suggest its possible self-activation and instead they suggest its activation through sigF for which we do not have any explanation. There is no information about what stops the transcription of *spoIIID*, and its negative regulation by Spo0F (Table 4) seems to reflect the need of the model to minimize the residual error.

As discussed above, transcription of SpoIVFB starts in the mother cell from a SigE promoter, and this is correctly captured by the model (Table 4). SpoIIID seems to negatively regulate part of the SigE regulon. The model predicts a negative regulation by SigK indicating the existence of some delay between the transcription of *spoIIID* and its regulatory role on SpoIVFB. Of the existing gene expression profiles, the best match to this functional profile seems to be that of *sigK*, and this is what the model predicts.

To be active, SigK requires its processing by SpoIVFB as the model suggests and SpoIVCB (one of the elements that make up SigK in *B. subtilis*) could be transcribed through a SigK promoter with the help of SpoIIID. The model captures this *sigK* self-induction but is not able to capture the effect of SpoIIID (Table 4). In *B. subtilis*, GerE negatively regulates the transcription of *sigK*. This is not captured by our results, however it is not clear if this negative regulation also happens in *B. anthracis*.

Transcription of *gerE* takes place from a SigK-dependent promoter and it stimulates the transcription of some SigK-dependent genes while inhibiting others. The importance of SigK has been correctly captured in our solution (Table 4). However the negative roles of *spoIVFB* and *sigG* cannot be explained.

**A8. Regulatory interactions by a Dynamic Bayesian Network based method using the sporulation data for *B. anthracis*.**

We wanted to compare the results obtained using our method with that from a related method that has already been proposed in the literature. As was mentioned in the Introduction section to the paper, Dynamic Bayesian Network (DBN) based methods are similar in principal to our method. So we obtained the regulatory interactions identified by a freely available software, BANJO [16] which employs a DBN based method using the sporulation data for the 9 genes of *B. Anthracis* that were considered in the paper.

The ‘maxMarkovLag’, ‘minMarkovLag’ parameters for this software were set to 1, parameter ‘maxParentCount’ was set to 3 and parameter ‘maxProposedNetworks’ were set to a million. The 9 gene expression variables were discretized to 5 levels. The results are shown in Table A.9 for the interactions which were common to the five best inferred networks..

**Table A.9**: Identified interactions obtained from the inference method for the set of 9 genes involved in the sporulation cascade on *B. anthracis*. ‘1’ indicates that there is likely to be an interaction. Whether these interactions are activating or inhibiting could not be conclusively verified by the method. ‘0’ indicates an absent interaction. A row corresponds to a regulated gene and a column corresponds to a regulator gene.

|  | *spo0F/Spo0A~P* | *sigF* | *sigE* | *spoIIIJ* | *sigG* | *spoIVFB* | *spoIIID* | *sigK* | *gerE* |
| --- | --- | --- | --- | --- | --- | --- | --- | --- | --- |
| *spo0F*/Spo0A~P | 1 | 0 | 0 | 0 | 1 | 0 | 0 | 1 | 0 |
| *sigF* | 0 | 1 | 0 | 0 | 0 | 0 | 1 | 0 | 1 |
| *sigE* | 0 | 0 | 1 | 0 | 1 | 0 | 0 | 1 | 0 |
| *spoIIIJ* | 0 | 0 | 0 | 1 | 0 | 0 | 0 | 0 | 1 |
| *sigG* | 0 | 0 | 0 | 1 | 1 | 0 | 0 | 0 | 1 |
| *spoIVFB* | 0 | 0 | 0 | 0 | 0 | 1 | 0 | 1 | 1 |
| *spoIIID* | 0 | 1 | 0 | 0 | 0 | 0 | 1 | 1 | 0 |
| *sigK* | 0 | 0 | 0 | 0 | 1 | 0 | 0 | 1 | 1 |
| *gerE* | 0 | 0 | 0 | 1 | 0 | 0 | 0 | 0 | 1 |

We observe that the method could not conclude whether any of the interactions that it identified were activating or inhibiting. Also, not many of the known interactions are identified when we compare the results with the known interactions shown in Figure A.2.

## A8. References

1. Horn RA, Hohnson CR: *Matrix Analysis*. NewYork: Cambridge University; 1999. Press. New York.
2. Paredes CJ, Alsaker KV, Papoutsakis ET: **A comparative genomic view of clostridial sporulation and physiology**. Nat Rev Microbiol. 2005, **3(12)**:969-78.
3. Liu H, Bergman NH, Thomason B, Shallom S, Hazen A, Crossno J, Rasko DA, Ravel J, Read TD, Peterson SN, Yates III J, Hanna PC: **Formation and Composition of the Bacillus Anthracis Endospore**. Journal of Bacteriology, 2004, **186(1)**:164-178.
4. Barrett T, Edgar R: **Gene expression omnibus: microarray data storage, submission, retrieval, and analysis.** Methods Enzymol. 2006, **411**:352-69.
5. Yang H, Haddad H, Tomas C, Alsaker K, Papoutsakis ET: **A segmental nearest neighbor normalization and gene identification method gives superior results for DNA-array analysis.** Proc Natl Acad Sci 2003,100(3):1122-7.
6. Tomas CA, Alsaker KV, Bonarius HP, Hendriksen WT, Yang H, Beamish JA, Paredes CJ, Papoutsakis ET: **DNA array-based transcriptional analysis of asporogenous, nonsolventogenic Clostridium acetobutylicum strains SKO1 and M5**. J Bacteriol. 2003, **185(15)**:4539-47.
7. MATLAB, MathWorks, Natick, MA, USA.
8. Sonenshein AL, Hoch JA, Losick R (Eds): *Bacillus subtilis* *and its closest relatives: From Genes to Cells*. Washington DC: ASM Press; 1970.
9. Errington J: **Regulation of endospore formation in Bacillus subtilis**. Nat Rev Microbiol. 2003 , **1(2)**:117-26.
10. Hilbert DW, Piggot PJ: **Compartmentalization of gene expression during Bacillus subtilis spore formation**. Microbiol Mol Biol Rev. 2004, **68(2)**:234-62.
11. Eichenberger P, Fujita M, Jensen ST, Conlon EM, Rudner DZ, Wang ST, Ferguson C, Haga K, Sato T, Liu JS, Losick R: **The program of gene transcription for a single differentiating cell type during sporulation in Bacillus subtilis**. PLoS Biol. 2004, 2(10):e328.
12. Craven P, Wahba G: **Smoothing Noisy Data with Spline Functions: Estimating the Correct Degree of Smoothness by the Method of Generalized Cross Validation**. Journal of Numerical Mathematics 1979, **31**:377-403.
13. Hambraeus G, von Wachenfeldt C, Hederstedt L: **Genome-wide survey of mRNA half-lives in Bacillus subtilis identifies extremely stable mRNAs**. Mol Genet Genomics. 2003, **269(5)**:706-14.
14. Varshavsky A: **The N-end rule: functions, mysteries, uses**. Proc Natl Acad Sci U S A. 1996, **93(22)**:12142-9
15. Voit EO: *Canonical Nonlinear Modeling – S-System Approach to Understanding Complexity.* New York: Van Nostrand Reinhold; 1991
16. Hartemink AJ: *Bayesian Network Inference with Java Objects (BANJO)*. 2005
